# Diversitäts- und kultursensible Gesundheitsinformationen für mehr digitale Gesundheitskompetenz: Eine kollaborative Community-Forschung zu Barrieren und Bedarfen

**DOI:** 10.1007/s11553-023-01012-z

**Published:** 2023-02-13

**Authors:** Anna Geldermann, Christiane Falge, Silke Betscher, Saskia Jünger, Caren Bertram, Christiane Woopen

**Affiliations:** 1 Cologne Center for Ethics, Rights, Economics, and Social Sciences of Health, Universität zu Köln, Köln, Nordrhein-Westfalen Deutschland; 2 Department of Community Health, Hochschule Gesundheit Bochum, Bochum, Nordrhein-Westfalen Deutschland; 3 Department Social Work, Hochschule Angewandte Wissenschaften Hamburg, Hamburg, Hamburg Deutschland; 4 Center for Life Ethics, Universität Bonn, Bonn, Nordrhein-Westfalen Deutschland

**Keywords:** Postmigration, Diversität, Online-Gesundheitsinformationen, Gesundheitskompetenz, Kollaboratives Forschen, Teilhabe, Postmigration, Diversity, Online health information, Health literacy, Collaborative research methods, Participation

## Abstract

**Hintergrund:**

Bei Gesundheitsfragen spielt das Internet eine zentrale Rolle, wobei Verbraucher:innen vor der Herausforderung stehen, geeignete Such- und Bewertungsstrategien zu entwickeln. Damit Informationen zur Gesundheit im Internet gefunden, verstanden, beurteilt und angewendet werden können, bedarf es digitaler Gesundheitskompetenz auf individueller und auf organisationaler Ebene. Vor dem Hintergrund gesellschaftlicher Pluralität und Diversität wurden Fähigkeiten und Zugänge marginalisierter Communities in diesem Zusammenhang bisher wenig beforscht.

**Ziel:**

Diese Studie untersuchte die Nutzung von Online-Gesundheitsinformationen im Alltag aus einer postmigrantischen Perspektive, welche nicht Migrationshintergründe, sondern lokale Kontexte von Migrant:innen in Deutschland als Vulnerabilitätsvariable versteht. Ziel dieser Studie war es, sowohl das digitale Gesundheitsinformationsverhalten marginalisierter Communities ethnografisch und kollaborativ zu erforschen als auch praxisnahe und kultursensible Ansätze für Akteur:innen der Gesundheitskommunikation zu entwickeln ohne migrantisierende Zuschreibungen zu reproduzieren.

**Methoden:**

Im Rahmen einer kollaborativen ethnografischen Feldforschung im Stadtteillabor der Bochumer Hustadt von 10/2020–01/2021 führten Community-Forscher:innen online leitfadengestützte Interviews in ihrem Umfeld durch. Die Interviews wurden mittels einer qualitativen Inhaltsanalyse ausgewertet.

**Ergebnisse und Diskussion:**

Die Ergebnisse machen deutlich, dass sprachliche, inhaltliche und mediale Aspekte von Online-Gesundheitsinformationen den Zugang zu und Umgang mit diesen für marginalisierte Communities bedingen. Die postmigrantische Perspektive stellt einerseits Migration als hinreichende Analyse- und Erklärungskategorie in Frage und begründet zugleich Diversitäts- und Kultursensibilität als zentrale Komponenten des Zugangs zu Gesundheitsinformationen und der Entwicklung von Maßnahmen zur Stärkung der digitalen Gesundheitskompetenz in pluralen Gesellschaften. Es bedarf sowohl vielsprachiger, transkulturell resonierender und technisch barrierearmer Online-Gesundheitsinformationen als auch gestärkter Interaktion zwischen Individuen einer pluralen postmigrantischen Gesellschaft und der Online-Gesundheitskommunikation.

## Abstract (en)

**Background:**

When answering health-related questions, consumers turn to the internet to find answers. However, finding health information online can be challenging. Therefore, consumers need to develop suitable search and evaluation strategies. Finding, understanding, assessing, and applying health information requires digital health literacy. Hence, digital health literacy concerns individual and organizational levels. Against the background of social plurality and diversity, the skills and conditions of accessibility of marginalized communities have so far received little attention in this context.

**Objectives:**

This study examines the use of online health information in daily life from a post-migrant perspective which considers the local contexts of migrants in Germany as a vulnerability variable rather than the migration histories. The aim of this study was to take a post-migrant perspective and to ethnographically and collaboratively study the digital health information behavior of marginalized communities as well as to develop practical and culturally sensitive approaches for actors in health communication.

**Methods:**

Collaborative ethnographic field research on information behavior regarding health-related questions was carried out online from 10/2020–01/2021 in the marginalized urban district of Bochum Hustadt, Germany. Researchers from the urban neighborhood conducted guided interviews in their communities. The interviews were analyzed using qualitative content analysis.

**Results and conclusion:**

The results indicate that language, content, and media aspects of online health information shape access to and handling of health information for individuals from marginalized communities. The post-migrant perspective challenges migration as a relevant category of analysis and explanation, while substantiating diversity and cultural sensitivity as central components of access to health information and the development of measures to strengthen digital health literacy in plural societies. There is a need for multilingual, transcultural, and technically barrier-free online health information as well as stronger interaction between individuals in a plural post-migrant society and online health communication.

## Hintergrund

Das Interesse an Gesundheitsinformationen ist groß – allgemeine sowie konkrete medizinische und gesundheitsbezogene Themen werden gezielt gesucht [39]. Das Internet spielt als Primärquelle eine durchaus wesentliche Rolle [4]. Vor diesem Hintergrund wurden im Rahmen des Projekts Orientierungshilfen im Umgang mit Gesundheitsinformationen im Internet (OriGes)^1^ die zwei Webseiten www.gesund-im-netz.net und www.klick2health.net als Orientierungshilfen und Wegweiser zum Umgang mit Online-Gesundheitsinformationen für Erwachsene und Jugendliche entwickelt. Diese sollen Nutzer:innen im Internet Hilfestellung und Tipps zum Umgang mit Online-Gesundheitsinformationen geben sowie exemplarisch konkrete Hinweise zur Recherche nach verlässlichen Informationsangeboten bieten.

Im Folgenden ersten Teil werden für diese Studie wesentliche Konzepte und Perspektiven kurz vorgestellt: Medien in komplexen Gesellschaften, (digitale) Gesundheitskompetenz als interaktionales Modell und theoretischer Rahmen sowie die postmigrantische Perspektive als gesellschaftstheoretische Grundannahme, welche der gesamten Studie zugrunde liegt. Im zweiten Teil werden die Methoden und das Forschungsdesign der Community-Forschung beschrieben. Im dritten Teil werden die Ergebnisse vorgestellt. Diese werden im abschließenden vierten Teil vor dem Hintergrund der Zugänge marginalisierter Gruppen zu Online-Gesundheitsinformationen sowie einer diesbezüglichen Weiterentwicklung von Gesundheitswebseiten diskutiert.

### Medien und komplexe Gesellschaften

Alltags- und Lebenswelten rund um Gesundheitsfragen werden im Kontext des Internets und *Dr. Google* durch Medien beeinflusst. Kultur- und Gesellschaftsprozesse werden dabei weitgehend von Medien und deren Logiken geprägt. Insbesondere die Kommunikation ist nicht nur zunehmend von Medien geprägt, sondern auch davon abhängig. Dieser Prozess vollzieht sich sowohl durch die Weiterentwicklung von Hard- und Software-Technologien als auch durch den Wandel in Mediensystemen beispielsweise der Presse oder Social Media [24]. Daher kann von einer Mediatisierung der Lebenswelten gesprochen werden. Effekte und Potenziale mediatisierter Lebenswelten sind insbesondere mit Blick auf die Kommunikation und Wirklichkeitskonstruktion in Gesellschaften näher zu betrachten, da sie dort nahezu alle Kommunikationsprozesse betreffen und gestalten [23]. In dieser Studie wird ein weiter Medienbegriff verwendet, der Medien als sozialhistorisch bedingte Gesamtmediensysteme versteht, welche sowohl selbstorganisatorisch als auch koproduktiv zusammenwirken. Dabei wirken sich Medien auch auf die Entfaltung von Gruppen oder Gesellschaften aus [40, 44].

Die Kommunikation von Gesundheit bezieht sich nicht nur auf spezifische Krankheiten, sondern auch auf ganzheitliche Prozesse und damit auf die „[…] Vermittlung und den Austausch von Wissen, Meinungen und Gefühlen zwischen Menschen, die als professionelle Dienstleister oder Patienten/Klienten [sic] in den gesundheitlichen Versorgungsprozess einbezogen sind, und/oder [sic] als Bürgerinnen und Bürger an Fragen von Gesundheit und Krankheit und öffentlicher Gesundheitspolitik interessiert sind“ [17, S. 11].

Dieser Studie liegt ein postmigrantisches Forschungsparadigma zugrunde. Dieses stellt Migration als hinreichende Analyse- und Erklärungskategorie in Frage und nimmt zugleich Diversitäts- und Kultursensibilität^2^ als zentrale Komponenten des Zugangs zu Gesundheitsinformationen in den Blick [10]. Nicht die Differenzen in den Kompetenzen und Umgangsformen einzelner Bevölkerungsgruppen im Kontext von Online-Gesundheitsinformationen sind von Interesse, sondern soziale und mediale Rahmenbedingungen des Umgangs von marginalisierten Communities in spezifischen Bezugsräumen. Der Community-Ansatz dieser Studie fußt auf einem konstruktivistischen Kulturverständnis und berücksichtigt sowohl Potenziale handlungsfähiger und selbstbestimmter Individuen innerhalb einer Gruppe als auch die Heterogenität von Kollektiven [45]. Zugehörigkeiten und Praxis von Gruppen werden nicht als statisch betrachtet, sodass Kultur als konstruierbare und damit veränderbare Praxis prozesshaft verstanden wird [45, 46]. Es gilt die Gesundheitskommunikation hinsichtlich der Mediatisierung dort kritisch zu hinterfragen, wo Ungleichbehandlung und Marginalisierung von Gruppen durch mediale Kommunikationsprozesse entstehen, wo diese auf bestehende gesellschaftliche Ungleichheitsstrukturen treffen oder diese verstärken.

### Gesundheitskompetenz: zum Umgang mit Gesundheitsinformationen (im Internet)

Das Internet als Medium und Informationsraum ist durch verschiedene informationstechnische und algorithmische, politische, kulturelle, soziale sowie ökonomische Logiken strukturiert. Nutzer:innen des Internets sehen sich bei der Recherche nach Gesundheitsinformationen oftmals einer gewissen Undurchschaubarkeit ausgesetzt und müssen eigenständig sowohl Such-, als auch Bewertungsstrategien für Online-Gesundheitsinformationen entwickeln [3]. Insbesondere die Coronaviruspandemie und die anfängliche Informationsflut verdeutlichten die Relevanz effektiver Gesundheitskommunikation [34]. Die Bedeutung von Gesundheitskompetenz für den Pandemiealltag vieler Internetnutzer:innen stieg, denn es galt Gesundheitsinformationen zu finden, verstehen, beurteilen und für die eigene Gesundheitsversorgung und Lebensqualität anwenden zu können [34, 41]. Zudem spielt die individuelle und Community-bezogene Relevanz der Informationen und der Bezug zum Selbst eine nicht zu unterschätzende Rolle, denn Rezipient:innen können dann die gesundheitsrelevante Informationen kognitiv besser verarbeiten und erinnern, wenn sie Bezugspunkte zur eigenen Lebenswelt, Community und dem eigenen Selbst aufweisen. [42].

Im Rahmen der *Allianz für Gesundheitskompetenz* [8] soll diese in der Bevölkerung nachhaltig gestärkt und durch verschiedene Maßnahmen verbessert werden. Aktuelle empirische Studien zeigen jedoch weiterhin einen Handlungsbedarf [22, 32, 39]. Mehr als zwei Drittel der Internetnutzer:innen fällt es bei Gesundheitsfragen schwer, die Vertrauenswürdigkeit von Informationen einzuschätzen. Ebenso fällt es Nutzer:innen schwer zu beurteilen, ob hinter einer Information kommerzielle Interessen zu vermuten sind bzw. wie die für die eigene Situation passende Information gefunden und beurteilt werden kann [39].

Während Sørensen et al. [41] die individuellen Fähig- und Fertigkeiten im Rahmen der Gesundheitskompetenz in den Vordergrund rücken, gehen andere Definitionen von einer Relation zwischen dem Individuum und sozialen sowie strukturellen Kontexten aus [2]. Die Gesundheitskompetenz von Menschen wird auch auf organisationaler Ebene beeinflusst. Organisationen können es Individuen erleichtern, gesundheitskompetent zu handeln beispielsweise indem individuelle Bedürfnisse, Ressourcen und Fähigkeiten sowie das soziale Umfeld, Wertevorstellungen und Behandlungsziele bei der Entwicklung und Bereitstellung von Gesundheitsinformationen miteinbezogen werden. In diesem Zusammenhang wird von der „organisationalen Gesundheitskompetenz“ [6, S. 759] als zweite Wirkungsebene neben der individuellen Gesundheitskompetenz gesprochen. Wenn Gesundheitsthemen in komplexen mediatisierten Kommunikationssystemen verhandelt werden, der Umgang mit diesen Systemen jedoch durch konkrete individuelle Fähig- und Fertigkeiten bedingt ist, stellt dies Ansprüche an das Gesundheitssystem [41]. Aus diesem Grund plädiert Silja Samerski [38] für ein lebensweltorientiertes Konzept von Gesundheitskompetenz und kritisiert damit eine universalistische Konzeptualisierung von Gesundheitskompetenz. Auch in einem mediatisierten Gesundheitssystem, in welchem die Gesundheitskommunikation von Medien durchdrungen ist, sollten Menschen gestärkt und ermächtigt werden, in ihren individuellen Lebenswelten Gesundheitsinformationen finden, verstehen, bewerten und im Sinne ihrer Gesundheit anwenden zu können. Im Sinne der Befähigungsgerechtigkeit lässt sich dies mit Martha Nussbaum [31] auch als grundsätzlich geboten für das Funktionieren einer pluralen Gesellschaft argumentieren.

**Abb. 1:**
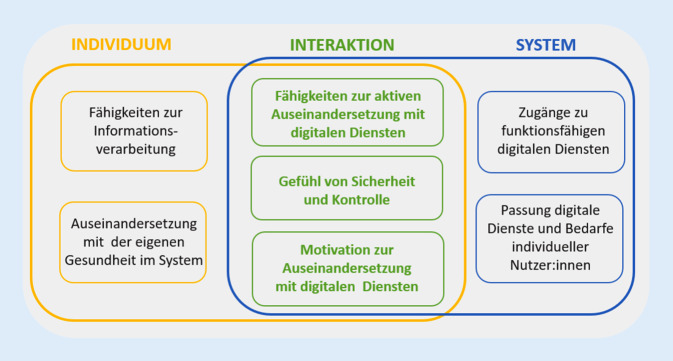
Modell zur digitalen Gesundheitskompetenz eHLF („e-health literacy framework“). (Norgaard et al. [29], eigene Darstellung)

Um den spezifischen Anforderungen im Umgang mit digitalen bzw. Online-Gesundheitsinformationen Rechnung zu tragen, wurde der Begriff *eHealth Literacy* oder zu Deutsch *digitale Gesundheitskompetenz *geprägt. Ausgehend vom Lilienmodell von Norman und Skinner [30] entwickelten Norgaard et al. [29] das „e-health literacy framework“ (eHLF). Der Ansatz integriert die individuelle Ebene von Gesundheitskompetenz in einem relationalen Gesamtkonzept der eHealth Literacy. Sie berücksichtigen insbesondere die Interaktion zwischen Individuen und der Kontextabhängigkeit von komplexen Systemen [29]. Somit hebt das Modell von Noorgard et al. die vom System geschaffenen Voraussetzungen und die Interaktion zwischen Individuen und dem System hervor (Abb. 1).

Nach Norgaard et al. [29] benötigen Individuen Fähigkeiten, um Informationen zu verarbeiten sowie die grundlegende Motivation, sich mit der eigenen Gesundheit im System auseinanderzusetzen. Diese von Norgaard et al. nicht weiter ausgeführten Fähigkeiten, welche Individuen für den Umgang mit Online-Gesundheitsinformationen brauchen, beziehen Kolpatzik et al. [22] in benanntem Zusammenhang auf 8 spezifische Dimensionen von Kompetenzen: (1) Computer, (2) Daten, (3) Datenschutz, (4) Lesen und Schreiben, (5) Medien, (6) Suchen und Finden, (7) Informationen und (8) Gesundheitsinformationen [22]. Zudem muss das System auf der anderen Seite für entsprechende Zugänge zu funktionsfähigen digitalen Diensten sowie einer Passung zwischen diesen Diensten und der Bedarfe individueller Nutzer:innen Sorge tragen [29]. Kolpatzik et al. [22] führen die Verantwortlichkeit des Systems weiter aus und sprechen sich für auf technischer Ebene niederschwellige Zugänge zu elektronischen Hilfsmitteln, zielgruppenspezifische Angebote und die Einbindung der Zielgruppe in die technische Entwicklung dieser aus. Weiterhin ist das System auf inhaltlicher Ebene für den Zugang zu evidenzbasierten Angeboten, der Berücksichtigung von Laienverständlichkeit sowie der Entwicklung von Maßnahmen zur Steigerung der Adhärenz verantwortlich. Dabei beeinflusst die Bereitschaft des Systems, sich an diese Bedarfe und Ressourcen anzupassen sowie letztlich der Grad der Anpassung die „perception of access“ [29, S. 533]. Durch die Interaktion zwischen Individuum und System kann die Motivation zur Auseinandersetzung mit der eigenen Gesundheit gefördert und ein Gefühl von Sicherheit und Kontrolle sowie die Akzeptanz der Angebote selbst gestärkt werden [22, 29].

In der Gesundheitskompetenzforschung werden verschiedene soziodemografische Daten vor dem Hintergrund des Konzepts der Vulnerabilität, im Sinne einer besonderen Verletzlichkeit, kategorisiert [37]. Beispielsweise wurde die Variable *mit Migrationshintergrund* als mit einer niedrigen Gesundheitskompetenz korrelierend beschrieben [39]. Die Kategorisierung von Migration als Vulnerabilitätsfaktor soll im folgenden Abschnitt aus einer postmigrantischen Perspektive mit Blick auf die Bedeutung und Beziehung der Konzepte *Diversität* und *Vulnerabilität* für die Forschungspraxis und Maßnahmenentwicklung hinterfragt werden.

### Eine postmigrantische Perspektive auf Gesundheitskompetenz

Der deutschsprachige Begriff *Diversität*, im Sinne von Verschiedenheit, bedeutet so viel wie Vielfalt beziehungsweise Pluralität. Der englischsprachige Begriff „diversity“ verweist stärker auf eine konzeptuelle Ebene von Diversität. Seinen Ursprung hat das Konzept der *Diversity* in der sozialkritischen Bürger- und Menschenrechtsbewegungen Schwarzer Menschen/„people of colour“, Frauen und der lesbisch, schwulen, bisexuellen, transgender, queer und intersexuellen* (LSBTQI*)-Communities. Als Konzept fokussiert *Diversity* in einer diskriminierungssensiblen Perspektive auf Zugangsmöglichkeiten und Teilhabe bzw. Ausschlüsse und Barrieren. Das Konzept „bezeichnet eine nicht von einem Einheitspunkt gedachte oder auf einen solchen bezogene Pluralität“ [43, S. 143]. Wenn Diversität als durch Differenzkonfigurationen erzeugt betrachtet wird, fußt dies auf der Konfiguration personenbezogener Unterschiede und der Annahme, dass Personen, Bevölkerungen, Organisationen und gouvernmentale Strukturen nicht nur in einen Gesellschaftsraum hineingeschrieben, sondern auch strukturell stabilisiert werden [28]. Dies wiederum hat Inklusions- und Exklusionsprozesse zur Folge. *Diversity*-Ansätze, welche auf der Anerkennung von Differenzen fußen, wurden aus sozialkritisch-feministischer Perspektive als ökonomisierende und neoliberale Ansätze [33], welche Machtverhältnisse und Ungleichheitsstrukturen zu wenig in den Blick nehmen, kritisiert und davon abgegrenzt. Mit der sozialkritisch-feministischen Konzeptualisierung von Diversität sollen künstliche und strukturelle Vereinheitlichungen und Ungleichheiten zugunsten der tatsächlich bestehenden Vielfalt auf kultureller, ethnischer, geschlechtlicher oder körperlicher Ebene aufgebrochen werden. Die Forderung und das Ziel des sozialkritischen Ansatzes ist es, Gleichbehandlung, Chancengleichheit und Diskriminierungsschutz sowohl auf gesellschaftlicher als auch auf organisationaler oder institutioneller Ebene für alle Menschen zu realisieren [26, 28, 33, 43].

Der einfache Transfer von *Diversity *auf verschiedene soziale Kontexte und gesellschaftliche Diskurse zeichnet sich durch gewisse Spannungen aus. So war beispielsweise im gesellschaftlichen Umgang mit Migration lange das Paradigma der *Integration*, im Sinne einer *idealen* Anpassung, vorherrschend [27]. Im Versuch dieses zu überwinden, prägte die Sozialwissenschaftlerin Naika Foroutan [11] den Begriff der *postmigrantischen Perspektive*. Diese Perspektive fokussiert auf die Pluralität von Gesellschaft und das Zusammenleben in einer solchen Gemeinschaft als Normalfall [27]. Zudem problematisiert sie die homogen gedachte Gesellschaft, in welcher Migration als ein kulturalistisch von der Norm abweichendes Phänomen betrachtet wird [12, 27]. Die postmigrantische Perspektive sieht Gesellschaft als durch vielfältige Migrationsbezüge geprägt. Gesellschaftserfahrung ist aus dieser Perspektive immer auch Migrationserfahrung und umgekehrt. Damit können alle Elemente von Gesellschaft als postmigrantisch gedacht werden [27]. Aus postmigrantischer Perspektive werden in der gesellschaftlichen Realität bestehende Narrative oder Kategorisierungen infrage gestellt und Alternativen entwickelt. Damit soll insbesondere das Denken der Kategorie *mit Migrationshintergrund* aufgelöst und durch „herkunftsübergreifende Erklärungen für gesellschaftspolitische Kernkonflikte um Anerkennung, Chancengerechtigkeit und Teilhabe in pluralen Demokratien“ [11, S. 271] ersetzt werden.

In der Migrationsforschung bezieht sich diese Kritik auch auf die ethnische Perspektive. Die Komplexität einer migrantischen Gesellschaft lässt sich nicht allein hinsichtlich ethnischer Heterogenität begreifen [7, 14]. Nicht nur im Feld der Migrationsforschung, auch in den Kultur- und Sozialwissenschaften führte dies zu einem heterogenen Verständnis von Gesellschaft, welches von transnationalen (Migrations)prozessen geprägt ist [7]. Damit rückte die Lokalität, insbesondere die urbanen Nachbarschaften, in den Forschungsfokus, was den Forschungsblick weitete, von der Kategorie Migrant:innen, hin zu Migrant:innen in prekären Kontexten [9, 13]. Diese prekären Kontexte können durch Ungleichheiten bezüglich sozioökonomischer sowie individueller Ressourcen Vulnerabilität konstruieren, wenn die umliegenden Systeme die Ungleichheiten nicht entsprechend berücksichtigen [15].

Wird Gesundheitskompetenz mit dem Ziel zu stärken beforscht, so stellen sich vor dem Hintergrund des gesellschaftstheoretischen Postulats der Postmigration neue Fragen an die Ansätze der Gesundheitskompetenzforschung sowie die Entwicklung von Gesundheitsinformationen. Islertas [20] hinterfragt, ob die Gesundheitskompetenzforschung einen offeneren Kulturbegriff brauche, welcher beispielsweise unterschiedliche Sprachkompetenzen angemessen in die Quantifizierung von Gesundheitskompetenz miteinbezieht, um ganzheitlichere, die gesamte Gesellschaft betreffende Konzepte zur Stärkung der Gesundheit zu entwickeln [19]. Denn die Sprache ist ein wesentlicher Teil von Kultur, welche wiederum Teil der Stärkung von Gesundheitskompetenz sein soll [20]. Für die Praxis der Gesundheitskompetenzforschung in einer postmigrantischen Gesellschaft gilt es folglich in dieser Studie sowohl Rahmenbedingungen des Umgangs mit Online-Gesundheitsinformationen einzelner Personen oder Communities zu betrachten, als auch über den verengenden analytischen Fokus *Migration* hinauszublicken. Damit rücken Faktoren wie beispielsweise Armut, Arbeitslosigkeit, benachteiligtes Wohnumfeld oder ein fehlender fester Wohnsitz sowie die Zugehörigkeit zu einer diskriminierten Minderheit, geringe Schuldbildung, fehlende Vertrautheit mit dem lokalen Gesundheitssystem, Sprachbarrieren oder soziale Isolation in den Vordergrund [9]. Diese Faktoren können jeweils bereits einen Einfluss auf den Umgang mit Online-Gesundheitsinformationen haben, sind jedoch häufig miteinander verknüpft, sodass sie sich gegenseitig verstärken können [15]. Vor diesem Hintergrund und in Anlehnung an die Definition von Grabovschi et al. [15] wird Vulnerabilität im Kontext dieser Studie als eine erhöhte Wahrscheinlichkeit verstanden, aufgrund einer Kombination unterschiedlicher individueller Faktoren (beispielsweise das Fehlen sozioökonomischer Ressourcen, soziale Eingebundenheit) und Umgebungsfaktoren (beispielsweise Bildungsinfrastrukturen, Zugang zum Internet, Sozialraum), Hürden im Umgang mit Online-Gesundheitsinformationen zu erleben. Damit wird auf die (über)generalisierte Kategorie der Migration/Ethnizität als Vulnerabilitätsfaktor und somit ätiologischer Faktor für eine verringerte Gesundheitskompetenz verzichtet [19].

Diese Studie lädt dazu ein, eine postmigrantische Perspektive für die Erforschung organisationaler und digitaler Gesundheitskompetenz einzunehmen. Dadurch können Ungleichheitsstrukturen, welche die Rahmenbedingungen des Umgangs mit Online-Gesundheitsinformationen marginalisierter Communities beeinflussen, fokussiert werden. Zudem steht in dieser Studie, neben der Berücksichtigung dieser Strukturen und vor dem Hintergrund der Konzeptualisierung von Diversität und der postmigrantischen Perspektive, die kulturelle Dimension von *Diversity* im Vordergrund, sodass im Folgenden insbesondere auf die Kultursensibilität im Kontext von Online-Gesundheitsinformationen fokussiert und diese begrifflich differenziert wird.

### Forschungsfragen

Ziel dieser Studie ist es, das gesundheitsbezogene Informationsverhalten der befragten Communities^3^ in den Blick zu nehmen und kultursensible Lösungsansätze für mehr Teilhabe an Online-Gesundheitsinformationen zu erarbeiten. Zudem soll die Bedeutung von soziokulturellen und medialen Rahmenbedingungen, welche den Umgang mit Online-Gesundheitsinformationen dieser Communities bedingen, untersucht werden. Die Forschung fand zu Beginn der zweiten Welle der Coronavirus Pandemie (Okt. 2020 bis Januar 2021) statt, die Pandemie spielte für den Analysefokus dieser Studie eine untergeordnete Rolle. Die übergeordneten Forschungsfragen lauteten: Wie sieht das gesundheitsbezogene Online-Informationsverhalten in der Bochumer Hustadt aus? Welche Zugänge und Zugangsbedingungen zu Online-Gesundheitsinformationen zeichnet die befragten Communities aus? Welche Implikationen für die Stärkung von Community-spezifischen digitalen Gesundheitskompetenzen lassen sich daraus ableiten?

## Methoden und Material

Die postulierte gesellschaftstheoretische Grundannahme der Postmigration spiegelt sich auch in der Methodologie und Methodik dieser Studie wider. Die Stichprobe setzt sich aus den Communities der Stadtteilforscher:innen des Stadtteillabors Bochum Hustadt zusammen. Es wurden keine weiteren Ein- oder Ausschlusskriterien bezüglich soziodemografischer Faktoren bestimmt. Entscheidend war der Bezug zur urbanen Nachbarschaft und zum sozialen Kontext der Stadtteilforscher:innen. Dadurch wurde eine äußerst heterogene Personengruppe im Verlauf des Projekts interviewt. Ein weiterer zentraler Aspekt der postmigrantischen Perspektive in dieser Studie war die Partizipation der Stadtteilforscher:innen selbst an der Datenerhebung, um Aspekte der Teilhabe an Online-Gesundheitsinformationen nicht *über*, sondern gemeinsam mit ihnen zu erheben. Somit dient die Studie nicht nur der ethnografischen Beschreibung des digitalen Gesundheitsinformationsverhaltens marginalisierter Communities, sondern beleuchtet soziale und mediale Rahmenbedingen des Umgangs mit Online-Gesundheitsinformationen, um Teilhabe an diesen für die Praxis in einer pluralen Gesellschaft fruchtbar zu machen.

### Community-Forschung: kollaborative ethnografische Feldforschung

Diese Forschung wurde in Kooperation mit dem Stadtteillabor Bochum Hustadt^4^ und der Hochschule für Gesundheit Bochum durchgeführt. Das Stadtteillabor ist ein im Jahre 2015 initiiertes Langzeitprojekt zur kollaborativen Gesundheitsforschung. Die Stadtgeschichte und -entwicklung Bochums ist vielseitig durch die Kohleindustrie und deren Kollaps geprägt. Ebenso prägten verschiedene Migrationsphasen die Stadtentwicklung, sodass sich Bochum heute durch höchst diverse urbane Strukturen auszeichnet. In der Hustadt leben ca. 4000 Menschen. Ungefähr 90 % der Bewohner:innen haben familiäre Migrationserfahrungen. Viele Bürger:innen der Hustadt leben von Sozialhilfe oder arbeiten im Niedriglohnsektor in mehreren Beschäftigungsverhältnissen gleichzeitig. Die Arbeit im Niedriglohnsektor ist oftmals mit vielen Arbeitsstunden und physisch herausfordernden Tätigkeiten assoziiert. Diese Arbeits- und Lebensbedingungen hinterlassen Spuren. Eine der häufigsten Erkrankungen im Quartier sind Bandscheibenvorfälle. Diskriminierungserfahrungen und eingeschränkte Bildungschancen sind ebenfalls Faktoren, welche das Leben und die Gesundheit der Hustädter:innen beeinflussen [9].

Das Stadtteillabor verbindet eine postmigrantische und medizinethnologische Perspektive mit einer kollaborativen Forschungspraxis und Methodologie, in der das Ziel des Forschens auf Augenhöhe ein zentrales Element darstellt. Im Stadtteillabor wird kollaborativ Wissen produziert, indem das Design von Fragebögen und die Befragungen selbst durch zuvor geschulte Community-Forscher:innen gestaltet werden [9]. Damit stellte das Stadtteillabor Bochum-Hustadt einen idealen strukturellen Rahmen für dieses Forschungsprojekt zum Umgang mit Online-Gesundheitsinfos im Kontext der kultursensiblen Erweiterung der Projektwebseiten www.gesund-im-netz.net und www.klick2health.net dar.

#### Schulungstage der Community-Forscher:innen

Im Rahmen dieser Studie wurden Bewohner:innen und Studierende der HS Gesundheit Bochum gemeinsam zu Community-Forscher:innen geschult. Die Ausbildung erfolgte innerhalb dreier Schulungstage im November und Dezember 2020. Das Ziel war es, in einem kollaborativen Prozess die Community-Forscher:innen dahingehend zu befähigen, dass sie selbstständig als Forscher:innen ins Feld gehen und leitfadengestützte Interviews in ihren Communities durchführen konnten. Am Projekt nahmen 23 Bewohner:innen der Hustadt in Bochum teil und 27 Studierende der HS Gesundheit. Die teilnehmenden Bewohner:innen erhielten eine Aufwandentschädigung von 12 €/h. Während der Schulungstage erarbeiteten die Community-Forscher:innen gemeinsam mit Studierenden spezifische Fragestellungen sowie einen entsprechenden Leitfaden für die qualitativen Interviews.

#### Leitfadenentwicklung und Vorbereitung der Interviews

Die Inhalte des Interviewleitfadens wurden partizipativ entwickelt. Bei der Formulierung der Leitfragen unterstützen die erfahrenen Wissenschaftler:innen die Community-Forscher:innen. Der Leitfaden bestand aus einem Vorfragebogen zu soziodemografischen Aspekten sowie Leitfragen zu diesen vier Themen: Digitales gesundheitsbezogenes Informationsverhalten, Orientierung bei Gesundheitsinformationen im Internet, Sprache, Wünsche und Bedürfnisse. Ein weiterer wichtiger Aspekt der Ausbildung der Community-Forscher:innen war die Einführung in die kollaborative Forschungspraxis und die Organisation der Interviews. Zudem wurde gemeinsam die Rolle und das eigene Verhalten als forschende Person reflektiert, über Funktionen und Ziele des Vorfragebogens gesprochen und die Anwendung von Leitfragen im Interview besprochen. Ebenfalls wurden Grundlagen ethischer Richtlinien und des Datenschutzes in der Forschung sowie wesentliche Bestimmungen zum Thema Anonymität und Pseudonymisierung vermittelt.

#### Rekrutierung, Durchführung der Interviews in den Communities und Reflexionstag

Es wurde ein erster Forschungstag im Dezember 2020 durchgeführt, an dem die Community-Forscher:innen online leitfadengestützte Interviews in ihren Communities führten. Die Community-Forscher:innen organisierten diese eigenständig und klärten die Teilnehmenden über die Studie und die anschließende Datenverarbeitung mündlich auf. Die Aufnahmen der Interviews erfolgten im Einvernehmen der Interviewpartner:innen. Die Interviews wurden abhängig von Präferenzen und Sprachkompetenzen in der deutschen Sprache oder der jeweiligen Muttersprache der Bewohner:innen geführt. Auf Arabisch, Kurdisch, Russisch oder Somali geführte Interviews wurden eigenständig von den Community-Forscher:innen mit entsprechenden Sprachkenntnissen übersetzt. Anschließend wurden von allen Interviews deutschsprachige Transkripte nach Kuckartz [25] angefertigt. Die Stärke der sprachlichen Glättung hing dabei von der Interviewsprache und den Sprachkompetenzen der Beteiligten ab.

### Datenanalyse

Insgesamt wurden 46 Interviews geführt und transkribiert. Die Interviewten gaben im Rahmen des Vorfragebogens Auskunft über soziodemografische Angaben. Die Tonaufnahmen wurden über die Community-Forscher:innen im Rahmen der Transkription archiviert und eigenverantwortlich gelöscht. Die Transkripte wurden DSGVO-konform archiviert und werden nach spätestens 10 Jahren gelöscht. Es wurden 27 von den 46 Interviews in die Analyse eingeschlossen (Tab. 1). Von den eingeschlossenen Interviews lagen jeweils soziodemografische Daten und Transkripte des Interviews vor. Die Länge der Interviews betrug zwischen 7 min und mehr als 1,5 h.

**Tab. 1:** Ein- und Ausschlusskriterien der Transkripte für die Inhaltsanalyse

Einschlusskriterien	Ausschlusskriterien
Ausgefüllter Vorfragebogen	Kein oder unzureichend ausgefüllter Vorfragebogen
Interviewführung nach Leitfaden	Interviewstruktur weicht stark vom Leitfaden ab

Bei den ausgeschlossenen Interviews fehlte bei 16 der Vorfragebogen, welches eine Interpretation der Angaben zu wesentlichen soziodemografischen Parametern unmöglich machte. Bei drei der ausgeschlossenen Interviews wurde das Interview unabhängig von den Fragen des Interviewleitfadens geführt, sodass diese Interviews ebenfalls nicht eingeschlossen wurden. Die transkribierten Interviews wurden anhand einer qualitativen Inhaltsanalyse mittels eines deduktiv-induktiven Kodierverfahrens nach Kuckartz [25] mit der Software MAXQDA 2020.4.2 (VERBI GmbH. Qualitative Datenanalyse mit MAXQDA. Software for Win and macOS. 2020; Berlin, Deutschland) durch 2 Mitarbeiterinnen unabhängig voneinander ausgewertet. Durch dieses Vorgehen konnte, ausgehend von der deduktiven Kategorienbildung anhand des Leitfadens und durch eine induktive Vertiefung, ein Kategoriensystem entwickelt werden (Tab. A.1). Das Kategoriensystem differenzierte u. a. zwischen Codes bezüglich der Informationssuche, Gesundheitsfragen und Suchanlässe, Vertrauenswürdigkeit von Gesundheitsinformationen, Verstehen und sprachlicher Zugang zu Gesundheitsinformationen sowie Wünschen und Bedarfen.

### Die befragten Communities

Aus der deskriptiven Analyse der soziodemografischen Fragebögen konnte entnommen werden, dass die Mitglieder der befragten Communities zu gut zwei Dritteln mit Vorerkrankungen leben. Häufig genannt wurden Diabetes, Schilddrüsenerkrankungen, Nierenerkrankungen, Migräne, Bandscheibenvorfälle, chronische Kopfschmerzen und chronische Hautkrankheiten. Knapp die Hälfte der Befragten ist für die Pflege oder Betreuung naher Angehöriger verantwortlich. So werden beispielsweise die Kinder, aber auch Eltern, Nachbarn und Geschwister sowie Partner:innen teilweise oder komplett betreut bzw. gepflegt. Berufstätigkeiten werden von 18 der 27 in die Analyse einbezogenen Befragten ausgeführt. Sie arbeiten beispielsweise in einer Imbissbude oder im Rahmen von Aushilfsjobs in der Gastronomie oder im Hotelgewerbe. Die Hälfte der Befragten lebt seit bis zu zehn Jahren in Deutschland, knapp ein Viertel aber auch seit mindestens 16 Jahren. Knapp die Hälfte der Befragten identifiziert sich als kurdisch, knapp zwei Drittel der Befragten sind im Irak oder Syrien geboren. Knapp ein Drittel der Befragten lebt zusammen mit den Eltern und ein Viertel mit Partner:innen und Kindern sowie ca. ein Fünftel ausschließlich mit Partner:innen zusammen (Tab. B.1).

## Ergebnisse: kultursensible Zugänge zu Online-Gesundheitsinformationen

Die Ergebnisse der qualitativen Inhaltsanalyse zeigen Community-spezifische Zugänge zu Online-Gesundheitsinformationen. Mehrsprachigkeit, eine *Resonanzbeziehung*^5^* mit Inhalten und Gestaltung* sowie Multimedialität und Barrierefreiheit werden als wesentliche, den Zugang zu Online-Gesundheitsinformationen bedingende Merkmale sichtbar. Diese Aspekte verdeutlichen die Rolle von Kultursensibilität in Bezug auf die Entwicklung und Gestaltung von Gesundheitsinformationen sowie Maßnahmen zur Stärkung von Gesundheitskompetenz in postmigrantischen pluralen Gesellschaften.

### Mehrsprachigkeit

Das Finden und Verstehen von Gesundheitsinformationen ist zunächst bedingt durch Sprachkompetenzen und durch das Literalitätsniveau in der jeweiligen Sprache, in welcher Gesundheitsinformationen gesucht werden. Für die Mehrheit der befragten Communities ist Deutsch nicht die Muttersprache, sondern Somali, Russisch, Arabisch oder Kurdisch. Die deutsche Sprache sowie das lateinische Alphabet sind für viele Personen eine später im Leben erlernte Fremdsprache und Schrift. In den befragten Communities wird sowohl auf Deutsch nach Gesundheitsinformationen gesucht als auch auf Arabisch, Kurdisch oder Englisch. Insbesondere Personen mit der Muttersprache Arabisch bevorzugen es, ausschließlich auf Arabisch nach Gesundheitsinformationen zu recherchieren, um medizinische Themen richtig zu verstehen. Dabei steht ihnen im Internet eine große Auswahl an Informationen zur Verfügung. Es werden Erklärvideos und YouTube-Kanäle arabischsprachiger Anbieter rezipiert.

„Ich weiß nicht, weil, normalerweise jeder sucht Informationen auf der Sprache die er beherrscht. […]. Aber auf Deutsch, wenn man hätte auf einfache Sprache, das würde bestimmt ich verstehen. Die Information, die da jetzt auf Deutsch stehen, das sind so auf höherem Niveau geschrieben. Die sind für Akademiker geschrieben bestimmt. Und die Leute, die wie ich verfügen über die B1-Niveau – bestimmt die können die Sprache nicht verstehen. […] Weil das Ziel von diesen Informationen ist, dass Leute das verstehen. Und natürlich, wenn das dann einfacher ist, das glaube ich, das für einen großen Teil wird das sehr sinnvoll sein.“ (IP18, Pos. 70)

Die eigenen Deutschkenntnisse bedingen die Recherchewege im Internet und können den Zugang zu und Umgang mit deutschsprachigen Informationen erheblich beeinträchtigen. Das Fehlen wichtiger Gesundheitsinformationen, insbesondere Informationen aus und über das deutsche Gesundheits- und Versorgungssystem in Fremdsprachen wird als Barriere wahrgenommen. Nichtsdestotrotz wird betont, dass Gesundheitsinformationen, die in einem deutschsprachigen Kontext stehen, versucht werden, in deutscher Sprache zu finden; so beispielsweise wenn Ärzt:innen gesucht werden.

„Weil ich in Deutschland lebe, manche Informationen soll ich auf den deutschen Seiten schauen, zum Beispiel, wenn es um Deutschland geht. Zum Beispiel, ich suche nach einem Arzt, welcher Arzt ist gut, welcher Arzt ist nicht gut, wie kann ich zu einem Arzt kommen. Solche Informationen ich suche auf Deutsch. Aber wenn es um Medizinisches geht, ich gucke auf unserer Sprache, auf Arabisch und Kurdisch.“ (IP17, Pos. 56)

Wenn die Interviewpartner:innen bei der Recherche nach deutschsprachigen Gesundheitsinformationen Sprachkompetenz bedingte Hürden erleben, lassen sich 4 Bewältigungsstrategien identifizieren: (1) Verständnisprobleme auf Sprachebene führen durchaus zum Abbruch der Recherche. (2) Andere suchen Unterstützung bei persönlichen Ansprechpartner:innen, wie der Sprachlehrer:in, Ärzt:innen oder Verwandten mit besseren Deutschkenntnissen, um sich die deutschsprachigen Inhalte zu erschließen. (3) Eine weitere Strategie ist die Recherche oder Übersetzung einzelner unbekannter Begriffe, bis der deutschsprachige Inhalt erschlossen werden kann. Alternativ wird auch der gesamte Textabschnitt übersetzt:

„Wenn ich etwas nicht verstehe, kopiere ich es und füge es in einem Übersetzerprogramm wieder ein und arbeite dann an der Übersetzung, vom Deutschen ins Arabische. Also wenn ich etwas nicht verstehe, übersetze ich es mir“ (IP11, Pos. 38).

Gerade mit Blick auf begrenzte zeitliche Ressourcen durch eine hohe familiäre oder Arbeitsbelastung wird ein solcher Übersetzungsprozess als mühselig empfunden. (4) Weiterhin führen diese Barrieren zu einem Wechsel der Recherchesprache, sodass Suchende direkt beispielsweise arabische oder persische Internetseiten aufsuchen. Dies wiederum kann zu Herausforderung führen, wenn die gefunden Informationen zum späteren Umgang damit in einen deutschsprachigen Kontext transferiert werden müssen. Zudem werden kostenfreie, oftmals über den Browser integrierte Übersetzungsprogramme wie Google Chromes Übersetzer verwendet.

Es wird angemerkt, dass es hilfreich wäre, mehrsprachige medizinische Versorgungsangebote, insbesondere für neu Zugewanderte konsultieren zu können.

„[…] dann finde ich ja, es sollte vielmehr Ärzte geben, wo man den Flüchtlingen wirklich so am Anfang, wenn sie nach Deutschland kommen, für jede Stadt, wahrscheinlich ist das Werbung man darf das wahrscheinlich auch nicht machen, ist vielleicht auch nur ein Wunschdenken von mir, dass man sagt: Hey du sprichst türkisch, arabisch, was auch immer, das sind folgende Ärzte, die sprechen auch diese Sprache. Da kannst du hingehen, weil es um deine Gesundheit geht. Es geht ja nicht darum, dass du dahin gehst, damit du deine eigene Welt baust, um nur mit Arabern unterwegs zu sein. Dafür bist du nicht nach Deutschland gekommen, aber das man sagt: Hey, wenn es um die Medizin geht, also da, wo du Unterstützung brauchst, weil du noch nicht der deutschen Sprache mächtig bist, geh dahin.“ (IP1, Pos. 48)

### Resonanzbeziehung mit Inhalten und Gestaltung

Das *sich angesprochen fühlen oder mitgemeint sein* durch und von Informationen steht in einer wesentlichen Beziehung zum Zugang zu und Umgang mit Online-Gesundheitsinformationen, insbesondere deren Bewertung und Anwendung. Den Befragten wurden im Interview Bilder zweier fiktiver Internetseiten von Arztpraxen vorgelegt. Die erste Webseite zeigt eine fiktive Arztpraxis mit ausschließlich weiß-deutschen männlichen Ärzten. Die zweite Webseite zeigt eine fiktive Praxis eines Teams von Ärzt:innen unterschiedlicher diversitätsbezogener äußerer Merkmale (Geschlecht, Hautfarbe, religiöse Symbole). Das zweite Bild wurde mehrheitlich präferiert. Es wird als ansprechender und vertrauenswürdiger eingeschätzt.

„Da die Menschen auf der Seite unterschiedlichen Gruppierungen zugehören, macht das den Eindruck, dass diese Menschen aufgrund der Zusammenarbeit mich mehr akzeptieren und verstehen würden als das Team auf der ersten Seite. Wenn Menschen aus unterschiedlichen religiösen, ethnischen oder kulturellen Zugehörigkeiten miteinander arbeiten, dann erweitert dies den Horizont dieser Menschen. Man teilt natürlich die Erfahrungen aus unterschiedlichen Gebieten mit einander. Daher würde ich sagen, dass mich eher die zweite Seite anspricht und ich mich dort mehr akzeptiert fühle.“ (IP9, Pos. 68–69)

Die bildlich dargestellte Vielfalt der Praxisbelegschaft wurde äußerst positiv wahrgenommen. So vermutet eine befragte Person, dass unabhängig von religiösen oder kulturellen Symbolen auf der zweiten Webseite, dort ausführlichere und hilfreichere Informationen zu finden sein könnten:

„Ich sehe es nicht aus dieser Perspektive, ob die Seiten von den verschiedenen Nationalitäten oder ob die Ärzte das gleiche Geschlecht sind. Wenn ich mich entscheide, dann wähle ich nach dem Inhalt. Ich habe das Gefühl, dass die rechte Seite mit den verschiedenen Nationalitäten mehr Informationen hat.“ (IP12, Pos. 77).

Die visuelle Repräsentation von gesellschaftlicher Vielfalt im medizinischem Versorgungssystem wird aus dieser migrantischen Perspektive als direkter Hinweis für die Qualität des Versorgungsangebots gelesen. Ein heterogenes Praxisteam vermittelt den Eindruck von inhaltlicher Breite bezüglich der verfügbaren Versorgungsleistungen bzw. Gesundheitsinformationen und letztlich einer besseren Stimmigkeit zwischen Patient:in und Ärzt:in. Die Identifikation mit dem Informationsangebot in der Bildsprache, aber auch der Wortwahl ist den Befragten wichtig. Bezüglich gendersensibler Sprache wird betont:

„[…] und gerade auf zum Beispiel, ähm, Homosexualität oder Gender bezogen, ist ja dann auch immer gut, wenn ähm, die Sprache dann in so genderfreundlicher Sprache zum Beispiel geschrieben wird, dann fühlt sich ja jeder angesprochen.“ (IP15, Pos. 86).

Nicht nur die Identifikation mit dem Informationsangebot hinsichtlich kultureller oder religiöser Aspekte auf der Bild- oder Sprachebene, sondern auch die thematische Passung zwischen Informationen und der Lebenswelt ist für die Befragten von Bedeutung. Die Themenauswahl und -darstellung von Gesundheitsinformationen im Internet wird als wichtiger Einstiegspunkt für die Auseinandersetzung mit den Informationen identifiziert. Themen und Inhalte, welche die Lebenswirklichkeit der Befragten widerspiegeln, erleichtern die Anschlussfähigkeit und Übertragbarkeit in das eigene Leben.

Um näher zu verstehen, welche Informationen für die Befragten im Alltag u. a. in der Muttersprache wichtig sind, wurden Auslöser für Recherchen im Internet zu Gesundheitsthemen näher betrachtet. Fast täglich sind im Alltagsleben verschiedene Gesundheitsfragen präsent, insbesondere in Richtung der Ursachenabklärung und Handlungsmöglichkeiten bei unbekannten Symptomen oder Schmerzen. Die Recherche im Internet liegt dabei besonders nahe, wenn Personen temporär keine Ärzt:innen konsultieren können oder nicht sicher sind, ob die erlebte Symptomatik ein Notfall ist. Des Weiteren sind Informationen zur Vor- und Nachbereitung von Gesprächen mit Ärzt:innen oder medizinischen Interventionen von großem Interesse. Zudem gibt es ein großes Interesse an einem gesunden Leben und Informationen zur Gesundheitserhaltung und Prävention. Tipps und Tricks für einen gesunden Alltag, welche ein Ausgleich zu der hohen Arbeitsbelastung sein können, sind gefragt. Unter den Befragten, gibt es einige Personen, die selbst oder deren nahe Angehörige, chronisch erkrankt sind. Unter den chronisch erkrankten Befragten gibt es ein besonderes Interesse an Informationen zur Weiterentwicklung von Therapien und Medikamenten, da bei den chronisch erkrankten Menschen der Eindruck einer hohen Selbstverantwortlichkeit besteht. Wird das Internet mit diesen Fragen konsultiert, sind Suchmaschinen die erste Anlaufstelle, dabei wird wiederholt und ausschließlich Google benannt.

Unabhängig von der Sprache erleben die Befragten es als schwer, Informationen rund um das Versorgungswesen in Deutschland, lokale Gesundheitsangebote und Informationen zur Krankheitsbewältigung im Alltag zu finden, von denen sie sich *angesprochen oder mitgemeint fühlen*. Die Daten zeigen, dass spezifische kultursensible Informationsbedürfnisse zum Versorgungswesen in Deutschland bestehen. Diese beziehen sich explizit auf Themen wie Präventionsmaßnahmen, Beratungsstellen und die Navigation und Rechtslage im Gesundheitswesen. Zum anderen wird deutlich, dass der lokale Bezug zu Gesundheitsinformationen wichtig für die Befragten ist, wobei das Internet vielmehr als ein Instrumentarium oder Vermittler zu lokalen Angeboten oder Beratungsstellen fungiert. Der persönliche Kontakt mit dem Gesundheitssystem und die Möglichkeit, Fragen und Anliegen im eigenen Tempo klären zu können, zeigen sich als geschätztes Instrument der Gesundheitskommunikation.

Zudem sind Informationen zur Krankheitsbewältigung in der eigenen Lebenswelt von spezifischem Interesse. So beschreibt eine Befragte bezüglich Informationen über Medikamente:

„Manchmal sollte es mehr Informationen über Medikamente geben. Ärzte versuchen teure Medikamente zu verschreiben, sodass man diese bei der Apotheke kauft. Dabei gibt es ähnliche Medikamente mit dem gleichen Wirkstoff, die günstiger sind. Darüber sollten die Menschen mehr informiert sein.“ (IP8, Pos. 53)

Im Falle von Arzneimitteln besteht ein Informationsbedürfnis nach Alternativen, wenn beispielsweise im Falle der Verschreibung von zu- oder selbstzahlungspflichtigen Medikamenten aufgrund von soziökonomischen Bedingungen günstigere Alternativen schlicht notwendig sind.

### Multimedialiät und Barrierefreiheit

Das Smartphone wird als zentrales Endgerät bei der Suche nach Gesundheitsinformationen im Alltag benannt. Zugleich bedeutet es einen ortsunabhängigen Zugang zu Informationen, da der Zugriff auch unterwegs möglich ist. Bevorzugt konsumierte Content-Formate sind Videos (beispielsweise auf der Plattform YouTube). Dabei waren Erfahrungsberichte oder Erzählungen von Influencer:innen von besonderem Interesse. Unter den arabischsprachigen Befragten wird beispielsweise der arabischsprachige Gesundheits-YouTube-Kanal *Grüner Apfel*^6^ als Quelle für Gesundheitsinformationen gern rezipiert und empfohlen.

Zudem werden Erklärvideos sowie Info-Grafiken zur Wissensvermittlung oftmals Texten bevorzugt, da diese als leichter verständlich wahrgenommen werden.

„Die Videos und Bilder sind besser verständlich und das ist für mich besser. Manchmal lese ich Texte, die mich dann zu den Videos und Bildern bringen. Ich bevorzuge Webseiten, die Videos und Bilder enthalten, da das verständlicher für mich ist.“ (IP11, Pos. 44)

Insgesamt spielen Bilder und audiovisuelles Material eine wichtige Rolle für den Konsum von Informationen bei den Befragten. Besonders hilfreich sind erklärende Grafiken oder Erklärvideos, die einen Text entweder ganz ersetzen oder konkret ergänzen. Bilder, ohne inhaltlichen Mehrwert, welche eher zur abstrakten Illustration dienen, werden explizit als wenig hilfreich beschrieben z. B. das Bild eines Kopfes neben einem Textabschnitt zum Thema Kopfschmerzen. Bevorzugt würden in diesem Fall visuelle Ergänzungen in Form von Informationsgrafiken zu Kopfschmerzen, welche beispielsweise Schmerzpunkte oder ähnliches benennen.

Von zentraler Bedeutung für viele Befragte, welche Deutsch als Zweit- oder Fremdsprache erlernt haben, ist die Einfache und zuweilen auch Leichte Sprache. Inhalte auf Textbasis werden ausgewählt und rezipiert, wenn sie verständlich aussehen.

Zur Information über die Coronavirus Pandemie spielt neben Online-Plattformen auch das Fernsehen eine wesentliche Rolle. Die Tagesschau als Nachrichtensender im Fernsehen wird oftmals als Informationskanal zum Thema Corona benannt. Auch Nachrichtensender aus dem Heimatland oder dem Heimatland der Eltern in der Muttersprache sind zudem wichtige Informationsquellen zur Pandemielage im In- und Ausland.

„[…] Meine Mutter ist gesundheitlich sehr krank und deshalb haben wir sehr viele Probleme zu Hause, weil wir vieles neu beachten müssen. Deshalb ich frage viel nach, wenn mal so eine Info rauskommt, dann gucke ich ganz genau: «was ist das?» und dann mein Papa und meine Mutter haben nicht viel Ahnung mit Nachrichten, deshalb ich mache die Nachrichten auf arabischen Kanal, in al-jazeera und al-arabya und auch auf kurdische Kanäle, damit meine Eltern genau erfahren, was ist da und was alles läuft. Auch, was es in Syrien läuft, weil, die ganze Familie wohnt dort. Weil die ganze Familie lebt dort – und wir haben so Mitleid mit denen, deshalb meine Eltern wollen ganz genau wissen, wieviel Fälle sind dort und dann was genau dort passiert. Wie ich gesagt habe, meine Eltern vor allem gucken die Nachrichten vor dem Fernseher, weil sie können das genau verstehen.“ (IP17, Pos. 4)

Diese Beschreibung macht die Herausforderungen und die Verantwortung Einzelner deutlich, welche mehrere Sprachen sprechen und Gesundheitsinformationen verschiedener nationaler Gesundheitssysteme oder Medien rezipieren (können). Insbesondere im Kontext der Coronavirus Pandemie galt es unterschiedliche Informationen zwischen der Informationslage und Situationen im Ausland und in Deutschland abzugleichen und nachzuvollziehen. Zudem mussten für den deutschen Kontext relevante deutschsprachige Informationen für Angehörige mit geringer ausgeprägten Sprachkenntnissen selbst übersetzt und im Einklang mit den zusätzlich aus dem Ausland rezipierten Gesundheitsinformationen erklärt werden. Weiterhin mussten teils voneinander abweichende gesundheitspolitische Aspekte miteinander vereint werden.

## Diskussion und Implikationen für die kultursensible Orientierungshilfe zum Umgang mit Online-Gesundheitsinformationen

Bei Gesundheitsfragen bietet das Internet den Befragten die Möglichkeit, aktiv einem Informationsbedürfnis durch eine konkrete selbstgesteuerte Recherche nachzugehen. Die Interaktion zwischen Individuen, welche Informationen suchen und finden sowie dem System, welches Gesundheitsinformationen im Internet anbietet, erweist sich aus Community-Perspektive insbesondere bedingt durch Mehrsprachigkeit, eine Resonanzbeziehung mit Inhalten und Gestaltung sowie Multimedialität und Barrierefreiheit. Diese Ergebnisse decken sich mit den Empfehlungen und strategischen Vorschlägen des Nationalen Aktionsplans Gesundheitskompetenz zur Stärkung der Gesundheitskompetenz in einer Gesellschaft der Vielfalt [1].

### Mehrsprachigkeit.

Es braucht ausführliche und explizite durch das deutsche Gesundheitssystem systematisch bereitgestellte Gesundheitsinformationen in Fremdsprachen im Internet. Dies gilt insbesondere für Sprachen, die von großen Teilen der in Deutschland lebenden Bevölkerung gesprochen werden. Der Bedarf an Mehrsprachigkeit sollte im Hinblick auf globale dynamische Migrationsbewegungen flexibel gedacht werden, sodass zentrale Gesundheitsinformationen auf beispielsweise Englisch, Türkisch, Arabisch, Ukrainisch/Russisch für die entsprechenden Communities erreichbar sind. In einer postmigrantischen vielsprachigen Gesellschaft ermöglicht dies mehr Teilhabe an Gesundheitsinformationen für Personen, welche in Deutschland leben und Deutsch als Fremd- oder Zweitsprache lesen und sprechen und sollte im Gesundheitssystem als grundsätzliche Voraussetzung für Teilhabe an Gesundheitsinformationen und Versorgung verfügbar gemacht werden.

### Resonanzbeziehung mit Inhalten und Gestaltung.

Es bedarf einer Sensibilisierung für die inhaltliche und gestalterische Passung bzw. Stimmigkeit zwischen Gesundheitsinformationen und Rezipient:innen. Die Identifikation mit Gesundheitsinformationen ([42]; *das Mitgemeint sein/sich angesprochen fühlen*) stellt einen bedeutsamen Aspekt der Informationsrezeption dar und erweist sich im Kontext von Migration als ein wesentlicher Bestandteil kultursensibler Gesundheitskommunikation und sollte in einer postmigrantischen Gesellschaft für möglichst viele Teile der Gesellschaft möglich sein. Auf inhaltlicher Ebene bezieht sich dies beispielsweise auf die Berücksichtigung von Pluralität innerhalb von Informationen durch die Anerkennung und das Kommunizieren kultureller oder wertebezogener sowie machtkritischer Vielfalt zu einem Thema. Auch die Bereitstellung und Sensibilisierung für Informationsbedürfnisse in Verbindung mit der Lebenswelt marginalisierter Communities spielen eine Rolle. Es bedarf Basisinformationen zu Ansprüchen, Unterstützungsmöglichkeiten im deutschen Gesundheitssystem, aber auch konkrete kultursensible Informationen zu Themen wie interkultureller und interreligiöser Pflege oder Ernährungsberatung sowie zur Krankheitsbewältigung und zu Therapiemöglichkeiten, welche an die sozioökonomische und kulturelle Lebenswelt von migrantisch marginalisierten Gruppen anschließen. Insbesondere auf gestalterischer Ebene sind Diversität in der Bildsprache, aber auch Genderidentität auf Sprachebene sehr wichtig und werden als äußerst positiv und als Merkmale für Qualität gesehen.

### Multimedialität und Barrierefreiheit.

Weiterhin braucht es strukturell multimediale und barrierefreie Gesundheitsinformationen im Internet. Grafiken und Videos sollten derart beschriftet oder untertitelt sein, dass automatische Übersetzungsprogramme und Vorlesefunktionen die Inhalte schnell ausgeben können. Da Informationen hauptsächlich auf dem kleineren Smartphone-Bildschirm rezipiert werden, ist die Textlänge und -darstellung unbedingt zu beachten. Einfachheit und Nutzbarkeit der Informationsangebote haben einen hohen Stellenwert bei der Auswahl von Informationsanbietern. Da Smartphones eine so zentrale Rolle bei der Recherche spielen, ist es absolut notwendig, Informationsangebote und Plattformen, deren Designelemente und Navigation, auf die mobile Anwendung auszurichten. Zudem werden Informationen in beispielsweise Leichter Sprache oder Einfacher Sprache sehr geschätzt, da die kurzen, einfachen Sätze, ohne viele Nebensätze und die Begriffserklärungen das Verstehen in einer Fremdsprache erleichtern. Aber auch Hyperlinkstrukturen zu weiteren Informationsangeboten können den Zugang und die eigene Recherche erleichtern.

### Potenziale Kultursensibilität

Durch die Möglichkeit zur Interaktion können die Motivation, sich mit digitalen Gesundheitsangeboten auseinanderzusetzen und die Akzeptanz für die Angebote selbst erhöht werden. Norgaard et al. [29] argumentieren, dass Ressourcen, wie Sprachkompetenzen, der Zugang zu Endgeräten, Lese- und Rezeptionsmöglichkeiten, kulturelle Identität oder Genderidentität, Weltanschauung und religiöse Orientierung die Basis sind, von welcher der Zugang zum System und letztlich Gesundheitskommunikation im Allgemeinen und Gesundheitsinformationen im Speziellen ausgeht [29]. Die Bereitschaft eines Systems, Diversitätssensibilität insgesamt und insbesondere Kultursensibilität mitzudenken und umzusetzen weist nicht nur darauf hin, wer eingeschlossen und angesprochen werden soll, sondern auch wer ausgeschlossen und nicht angesprochen wird.

**Abb. 2:**
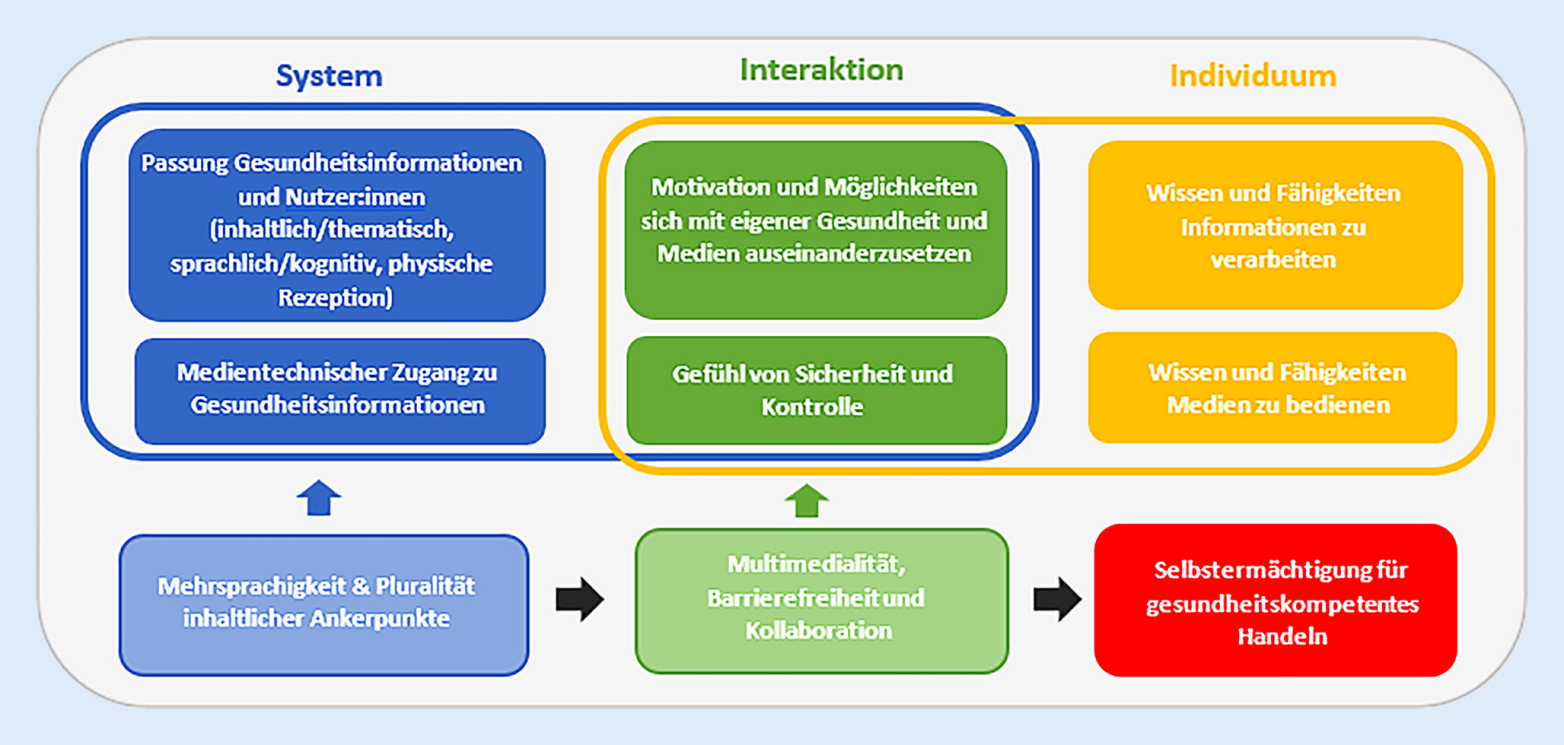
Kultursensibles Modell zur digitalen Gesundheitskompetenz eHLF („e-health literacy framework“). (Norgaard et al. [29], eigene Darstellung)

In Abb. 2 wird das Modell von Norgaard et al. [29] aus einer kultursensiblen Perspektive erweitert. Es ergänzt sprachliche Vielfalt und Resonanzräume mit kultursensiblen Inhalten als Verantwortlichkeit des Systems und Multimedialität sowie Barrierefreiheit als Modi der Interaktion, welche durch eine entsprechende Erweiterung gestärkt wird. Dies versetzt Individuen einer pluralen Gesellschaft perspektivisch in die Lage, Gefühle von Sicherheit und Kontrolle im Umgang mit Online-Gesundheitsinformationen zu entwickeln und Selbstermächtigung durch Gesundheitsinformationen zu erfahren. Somit werden Teile dieses interaktionalen Konzepts von Gesundheitskompetenz dahingehend spezifiziert, dass Diversität und Vielfalt vor dem Hintergrund von Pluralität nicht nur explizit berücksichtigt, sondern auch innerhalb des Modells integriert werden. Es wird deutlich, dass es einer Sensibilisierung und Verbesserung der Rahmenbedingungen von Gesundheitskommunikation im Kontext von Postmigration bedarf.

### Aufgaben zur Stärkung der Interaktion zwischen Individuen und dem Gesundheitssystem

Aus den Daten lassen sich zwei wesentliche Aspekte ableiten. Viele Befragte dieser Studie suchen und rezipieren Gesundheitsinformationen im Internet schlichtweg nicht in der deutschen Sprache und nutzen Informationsportale der Länder, die ihnen diese Informationen in ihrer Sprache zugänglich machen. Dieses Vorgehen ist, neben der Übersetzung von Informationen durch integrierte Übersetzungsanwendungen von z. B. Google Chrome, gängige Praxis und ist mit Blick auf den Zugang zu verlässlichen Informationen genauer zu betrachten. So wären beispielsweise die Qualität, Logiken und Maßstäbe der oftmals automatisch integrierten Übersetzungsprogramme kritisch zu hinterfragen, wenn es um die Übersetzung von Gesundheitsinformationen geht. Bei der Nutzung dieser oder ähnlicher Dienstleistungsangebote für die Internetrecherche, auch im Zusammenhang mit Suchmaschinen, knüpfen sich darüber hinaus Fragen rund um den Datenschutz bei der Suche nach teils sensiblen Gesundheitsthemen an.

Bezüglich der Recherche auf fremdsprachigen und ausländischen Informationsangeboten schließen sich Fragen zur Übertragbarkeit und Anwendbarkeit von beispielsweise Checklisten zur Qualität hinsichtlich der Evidenzbasierung von Gesundheitsinformationen an. Dies gilt es weiter hinsichtlich ihrer Potenziale und Herausforderungen zu untersuchen. Online-Gesundheitsinformationen sind unter anderem in kulturelle, strukturelle und mediale, normative oder politische Systeme eingebettet, sodass den Informationen verschiedene Gesundheitsnarrative beispielsweise bezüglich des Konzepts der Verantwortung innewohnen können, welche in einer postmigrantischen Gesellschaft von mehrheimischen Personen [47] in Anbindung an das deutsche Gesundheitssystem in Einklang gebracht werden müssen. Diesbezüglich gilt es in weiteren Forschungsvorhaben, sich insbesondere der Frage zu widmen, wie evidenzbasierte Online-Gesundheitsinformationen durch ein höheres Maß an Kultursensibilität inklusiver und verständlicher werden. Zu nennen wäre beispielsweise die Erarbeitung von Ansätzen für Gesundheitsinformationen rund um das Thema *Lebensende und palliative Versorgung*. Informationen zu diesen Themen gilt es insbesondere vor dem Hintergrund der Heterogenität spiritueller und kultureller Überzeugungen und Werten von Rezipient:innengruppen sensibel zu kommunizieren [21].

Zudem können Filterblasen das Recherchieren im Internet erschweren. Im Falle von politisch motivierten Filterlogiken oder gar Zensierungen von Internetseiten in der jeweiligen Sprache, stehen ans deutsche Gesundheitssystem angebundene Personen erneut vor der Herausforderung, die gewünschten Informationen zur Verbesserung, Erhaltung oder Widerherstellung der Gesundheit finden und bewerten zu müssen.

Im Folgenden wird auf zwei zentrale Lücken verwiesen, die aus der Forschung in Bezug auf digitale Gesundheitsangebote für marginalisierte Communities hervorgehen:

1. Es braucht eine proaktive Bekanntmachung und Aufklärung über Anbieter verlässlicher Online-Gesundheitsinformationen. Dazu bedarf es klarer und einfacher Kommunikation sowie mitunter zusätzlich offline Angebote durch beispielsweise vertraute Beratungsstellen, welche über Qualitätsmerkmale von Informationen, idealerweise in Fremdsprachen, aufklären. Vermittelte Qualitätsmerkmale sollten auf fremdsprachige und ausländische Gesundheitsinformationen angewendet werden können. Das Stadtteilforschungsprojekt QUERgesund [18], ein kassengefördertes kultursensibles Präventionsprojekt, dass im Co-Design mit den Hustadt Communities entstanden ist, [18] zeigt, dass insbesondere lokale, Community-spezifische und partizipative Angebote im Quartier richtungsweisende Ansätze hierfür sind. Im Rahmen dieses Projekts werden unter anderem Gesundheitsforen für Bewohner:innen zum Austausch, Lernen und Kennenlernen, digitale Yoga- oder Entspannungskurse, Frauenschwimmen oder ein Kurs zur gesunden Ernährung für männliche Jugendliche im Stadtteil angeboten. Themen der Gesundheitsforen sind beispielsweise Krankheiten verstehen oder verhindern, gesund bleiben oder Hilfe und Unterstützung kennenlernen. Die Bewohner:innen können diese Angebote in der Regel kostenfrei wahrnehmen.
2. Zudem braucht es systematisch strukturierte geeignete Rahmenbedingungen, durch welche Individuen Fähigkeiten im Umgang mit Online-Gesundheitsinformationen (digitale Gesundheitskompetenz) erlernen oder stärken können, damit Menschen in Deutschland im Internet solche Gesundheitsinformationen finden können, die für ihr Handeln relevant sind. Dabei gilt es die Medienkompetenz dahingehend zu stärken, dass Menschen trotz gesellschaftlicher, soziokultureller oder sozioökonomischer Marginalisierung Gesundheitsinformationen im Internet vor dem Hintergrund vielseitiger Zugangsmöglichkeiten, Nutzungsgewohnheiten und -präferenzen leichter rezipieren und beurteilen können. Konkrete Informationsangebote, didaktisch aufbereitete und lokal sowie offline begleitete Elemente sollten die Bedeutung und Tragweite von Informationen aus beispielsweise *Open-source-Portalen* vermitteln oder über die Logiken und Mechanismen einer Suchmaschinenrecherche aufklären und konkrete Handlungsmöglichkeiten aufzeigen. Ein Beispiel für die Stärkung von sowohl Medienkompetenz als auch Gesundheitskompetenz ist ein auf dieser Studie aufbauendes Folgeprojekt, im Rahmen dessen kollaborativ Informations- und Erklärvideos zu Gesundheitsthemen marginalisierter Communities gemeinsam mit Wissenschaftler:innen, Medienpartner:innen und Community-Forscher:innen entwickelt wurden^8^.

Den Gedanken der *Mediatisierung *[23] des Gesundheitskommunikationssystems aufgreifend, zeigen diese Daten die Bedeutung von Mediensystemen für die Entfaltung und Entfaltungsmöglichkeiten von Community-spezifischen Wirklichkeits- und Identitätskonstruktionen. Vor dem Hintergrund eines Medienbegriffs, welcher Medien als sowohl selbst organisierende als auch gemeinschaftlich produktiv zusammenwirkende Systeme versteht, wird deutlich, wie sich die derzeitige mediale Gesundheitskommunikation auf marginalisierten Gruppen auswirken kann. Im Internet, als Medium, sind Gesundheitsinformationen eingebettet in einen globalen und vernetzten Kontext. Gleichzeitig entgrenzt das Internet Gesundheitsinformationen als Medien auf raum-zeitlicher, nationaler und sozialer Ebene und erschafft neue Netzwerke. Diese können dann die Wahrnehmung der Wirklichkeit prägen. In einer solchen mediatisierten und postmigrantischen Gesellschaft können neue Gewohnheiten, Normen sowie Erwartungen entstehen. Diese stehen in einer Beziehung zu Gesundheitsinformationen im Internet, wenn Nutzer:innen sich von Online-Gesundheitsinformationen der *deutschen *„Dominanzkultur“ ([35]; sowohl sprachlich als auch kulturell) ausgeschlossen fühlen oder keinen Zugang dazu finden. Auch Baumeister et al. [5] argumentieren, dass es bezüglich erlebter Hürden in der Gesundheitskommunikation im Kontext von Migration weniger um die Migration oder andere konkrete Vulnerabilitätsmerkmale ginge, sondern darum, dass bestehenden Hürden vielmehr wie ein Brennglas Missstände im Gesundheitssystem verdeutlichen, die letztlich alle Menschen betreffen. Es wird deutlich, dass Angehörige unterschiedlicher nicht-deutsch-muttersprachlicher Communities sich entweder umständliche Umwege erarbeiten müssen oder auf fremdsprachige Informationen zugreifen, weil sie die Informationen des *deutschen* Gesundheitsinformationssystems nicht rezipieren können. Verschiedenste Formate, wie Videos oder Podcasts, die im Internet Gesundheitsinformationen auditiv und visuell vermitteln, ermöglichen es auf technischer, struktureller und konzeptioneller Ebene, Inhalte kultursensibel zu gestalten. Diversität in der Entwicklung von Gesundheitsinformationen mitzudenken obliegt wiederum dem Gesundheitskommunikationssystem.

## Limitationen und Reflexion

In dieser Studie wurden Interviewpartner:innen über das in der Bochumer Hustadt situierte Stadtteillabor und bestehende Netzwerke im Quartier rekrutiert. Ziel war die Rekrutierung von Personen aus marginalisierterten Stadtteilen. Interessierte Bewohner:innen wurden ausschließlich nach dem Einschlusskriterium Alter ≥ 18 Jahre aufgenommen. Die Methodologie dieser Studie sieht vor, dass die Community-Forscher:innen ihre Interviewpartner:innen selbst auswählen. Daher wurde seitens der Studienleiter:innen kein Einfluss auf das Sampling der Befragten genommen. Zudem bestehen möglicherweise Abweichungen in den deutschsprachigen Übersetzungen der Interviews, wenn die Übersetzung von nicht-deutschsprachigen Muttersprachler:innen vorgenommen wurde. Die Auswertung der soziodemografischen Daten weist darauf hin, dass das Sample der Befragten überwiegend aus muslimischen Befragten mit eigener Migrationserfahrung aus dem Mittleren Osten bestand. Über die Kooperation mit dem in der Hustadt situierten Stadtteillabor wurden in dieser Studie ausschließlich Personen aus marginalisierten Stadtteilen und keine Vergleichsgruppe aus beispielsweise privilegierteren Stadtteilen befragt. Ausgehend vom erhobenen Datenmaterial wurden in dieser Studie folgende Dimensionen von Diversität in der Analyse berücksichtigt: (soziale und nationale) Herkunft und Ethnizität, Kultur und Sprache. Daher wurden Dimensionen wie Behinderung, Alter, Religion und Weltanschauung oder Gender bei der Analyse ausgeklammert. Eine weitere Erforschung dieser Dimensionen von Diversität soll ausdrücklich ermutigt werden. Zudem erweist sich die Dimension des Alters als eine zentrale in diesem Zusammenhang weiter zu untersuchende, da sich im Verlauf der Forschung herausstellte, dass sowohl unterschiedlich mediatisierte Lebenswelten als auch generationsbedingte Affinitäten im Umgang mit Medien im Fragebogen nicht explizit berücksichtigt wurden.

In dieser Studie wurde zunächst eine Qualitative Inhaltsanalyse [25] durchgeführt, um erste latente und manifeste Themen (Barrieren und Bedarfe) der Befragten im Umgang mit Online-Gesundheitsinformationen zu erfassen, ordnen und strukturieren. Diese konnten mit bestehenden theoretischen Ansätzen und vor dem Hintergrund empirischer Daten zum Umgang mit Online-Gesundheitsinformationen diskutiert werden. Nichtsdestotrotz erlaubt gerade das aus der Ethnologie und Anthropologie stammende Forschungsparadigma hermeneutische Analyseansätze, um das implizite Wissen der Erfahrungswelt der Befragten im Umgang mit Online-Gesundheitsinformationen zu verstehen. Ein solcher Ansatz kann in einer Folgeforschung tiefere Einblicke in das Community-spezifische Informationsverhalten bei Gesundheitsfragen ermöglichen. In weiteren kollaborativen Untersuchungen können sowohl spezifische Maßnahmen für kultursensible Gesundheitsinformationen als auch diversitätssensible Gesundheitsinformation für Gruppen hinsichtlich weiterer Diversitätsdimensionen erschlossen werden. Es besteht weiterer Forschungsbedarf hinsichtlich der Nutzung nicht-deutscher Systeme und Kooperationen zwischen Systemen in beispielsweise transnational ausgerichteten Versorgungsansätzen oder Kooperationen zwischen Ärzt:innen und Patient:innen, die in transnationalen Lebenswelten leben.

## Fazit für die Praxis

- Es galt zu zeigen, wie der alltägliche Umgang marginalisierter Communities durch sprachliche, inhaltliche und mediale Aspekte von Online-Gesundheitsinformationen bedingt wird.
- Die Postmigration als gesellschaftstheoretischer Unterbau dieser Studie begründet das systematische Hinterfragen der Aussagefähigkeit von *Migration* als Analysekategorie und eine Öffnung für andere, Ungleichheit aufzeigende Analysekategorien sowie die Notwendigkeit kultursensibler Online-Gesundheitsinformationen.
- Für die Schnittstelle zwischen der sozialkritisch-feministischen Konzeptualisierung von *Diversität *und der Stärkung digitaler Gesundheitskompetenz bedarf es sowohl vielsprachiger, transkulturell resonierender und zugänglicher Gesundheitsinformationen als auch gestärkter Interaktion zwischen mehrheimischen Individuen und Online-Gesundheitskommunikation.

## Anhang A

### Kategoriensystem

**Tab. A.1:** Liste der Codes (MAXQDA)

Liste der Codes	Memo
**IN Informationssuche**	Überkategorie Suchverhalten. Umfasst Informationen zur Informationssuche im Alltag bei Gesundheitsfragen
*IN_M Online/Offline Medien*	Umfasst Informationen zum Suchverhalten in Online/Offline Medien, welche Verbraucher:innen beim Suchen und Finden von Gesundheitsinformationen nutzen
IN_M Internet allgemein	–
IN_M Webseiten	–
IN_M Social Media	–
IN_M Suchmaschinen	–
IN_M Rundfunk	–
IN_M Content-Formate	–
IN_M Endgeräte	–
IN_M Apps	–
IN_M Printmedien	–
*IN_PA Persönliche Ansprechpartner:innen*	Umfasst Informationen über persönliche Ansprechpartner bei Suche nach Gesundheitsinformationen. Auf welche Personen greifen die Verbraucher:innen bei Gesundheitsfragen zurück?
IN_PA Ärzt:innen und Versorgungswesen	–
IN_PA Familie/Freunde/Vertraute	–
IN_PA Vertraute mit med. Kenntnissen	–
*IN_PLA Priorisierungslogiken Ansprechpartner:innen/Medien*	Umfasst Informationen zur Priorisierungslogik der Ansprechpartner:innen bei Gesundheitsfragen. Wie ist die Reihenfolge der medialen oder persönlichen Ansprechpartner:innen (Hierarchie)?
*lN_S Suchumfang*	Umfasst Informationen zum zeitlichen und inhaltlichen Suchumfang bei Gesundheitsfragen. In welchem Umfang wird gesucht (zeitlich – Länge der Suche, Häufigkeit, inhaltlich – Informationstiefe bzw. Informationsdichte – wie viele verschiedene Quellen werden gesucht)?
*IN_U Umgebung Suche*	Umfasst Informationen zur Suchumgebung. Wo befinden Sie Verbraucher:innen bei der Suche, von welchen Orten aus starten sie die Suche?
**GSI Gesundheitsfragen und Suchanlässe Internet**	Überkategorie Gesundheitsfragen und Suchanlässe. Was sind Themen, Kontexte oder Suchanlässe der Verbraucher*innen? Warum suchen Verbraucher:innen nach Gesundheitsinformationen?
*GSI_U Unerreichbarkeit von Hausärztlicher Versorgung*	–
*GSI_A Vor- und Nachbereitung Arztbesuch/Intervention*	–
*GSI_K Kontakte/Ansprechpartner:innen im Versorgungssystem*	–
*GSI_L Gesundes Leben*	–
*GSI_CHRE Leben mit chronischer Erkrankung*	–
*GSI_S Unwohlsein/Symptomatik*	–
*GSI_COR Informationen rund um Corona/COVID-19*	–
**VG Vertrauenswürdigkeit von Gesundheitsinfos**	Überkategorie Vertrauenszuschreibung. Wie wird die Vertrauenswürdigkeit von Gesundheitsinformationen anhand welcher Kriterien und Strategien bewertet?
*VG_Z Zuschreibung Vertrauenswürdigkeit Gesundheitsinfos*	Welchen Angeboten vertrauen Verbraucher:innen? Wie sind Angebote beschaffen, die als vertrauenswürdig eingestuft werden?
VG_Z Gesundheitsinfos in (Social)Media	–
VG_Z Trefferliste Suchmaschine/Open Source	–
VG_Z Amtliche Seite/öffentliche Institutionen	–
VG_Z Seiten von Ärzt:innen	–
*VG_BS Beurteilungsstrategie Vertrauenswürdigkeit/Qualität*	Wie beurteilen Verbraucher:innen die Qualität von Gesundheitsinformationen?
VG_BS Keine feste Seite/Strategie	–
VG_BS Reflexion Urheber/Quelle der Informationen	–
VG_BS Intuition/Bauchgefühl/persönliche Sinnhaftigkeit	–
VG_BS Trial and Error/eigene Erfahrungen	–
VG_BS Vergleich/Abgleich mehrerer Seiten	–
VG_BS Rücksprache mit Vertrauten	–
VG_BS Rücksprache mit Ärzt:innen/med. Personal	–
VG_BS Bewertungen/Kommentare anderer User	–
*VG_M Begründung Misstrauen*	Wann und warum werden Verbraucher:innen gegenüber Online-Gesundheitsinfos misstrauisch?
VG_M Thematische/Inhaltliche Ausrichtung/Gestaltung	–
VG_M Zu viele/zu wenige Informationsangebote/Meinungen	–
VG_M Internet als Informationsraum	–
VG_M Unklare Qualität und Seriosität	–
**S Verstehen/sprachlicher Zugang Gesundheitsinfos**	Überkategorie Verstehen und sprachlicher Zugang Gesundheitsinformationen. Wie wird wo und in welcher Sprache recherchiert?
*S_R Recherche/Rezeptionssprache*	In welcher Sprache suchen Verbraucher:innen nach Gesundheitsinformationen?
S_R Mehrsprachige Recherche	–
S_R Arabisch	–
S_R Deutsch	–
*S_B Barrieren/Verständnisprobleme Fachbegriffe/Sprachkenntnisse*	Was sind und wodurch entstehen Verständnisprobleme?
S_B Sprachkenntnisse Deutsch	–
S_B Fachbegriffe	–
*S_U Umgang mit Barrieren*	Wie wird mit Barrieren umgegangen? Wie behelfen sich Verbraucher:innen, um Verständnisprobleme zu lösen?
S_U Abbruch der Recherche	–
S_U Aufsuchen Persönliche Ansprechpartner:in	–
S_U Fortsetzung Recherche/Recherche einzelner Begriffe	–
S_U Übersetzung/Einsatz anderer Sprache	–
S_U Fortsetzung Recherche/Recherche einzelner Begriffe	–
**W Wünsche und Bedarfe an Gesundheitsinformationen**	Überkategorie Wünsche und Bedarfe Gesundheitsinformationen. Welche Wünsche an Gesundheitsinformationen werden benannt?
*W_G Informationsgestaltung/Vermittlung Medienformatierung*	Wie sollten Informationen gestaltet (Aufbau, Struktur, Ästhetik, Format) sein, damit Verbraucher:innen sich angesprochen fühlen und sie verstehen?
W_G Informationsqualität/Vertrauen	–
W_G Schneller/kurzer Zugriff	–
W_G Einfache/Verständliche Formulierungen	–
W_G Mehrsprachigkeit/Sprachliche Vielfalt	–
W_G Resonanzbeziehung mit Inhalten und Gestaltung	–
W_G Medienformate/mediale Vermittlung	–
W_G Medienformatierung	–
*W_I Informationsbedarfe inhaltlich*	Zu welchen Themen wünschen Verbraucher:innen sich mehr Informationen?

## Anhang B

### Stichprobe

**Tab. B.1:** **Soziodemografische** Daten der Befragten (*n* = 27)

ID	Geschlecht	Geburtsland/Zugehörigkeit	Alter (Jahre)	Leben außerhalb des Geburtslands (Jahre)	Wohnsituation	Ausbildung^a^	Arbeitssituation
IP_1	Männlich	Syrien, kurdisch	35	5	Haushalt mit Frau & 3 Kindern	Grundschule (1–4), Klasse 5–8, Klasse 11–13, Universität; Hochschulabschluss als Arabischlehrer	In Ausbildung
IP_2	Männlich	Syrien, kurdisch	26	6	Lebt alleine in einem Haushalt	Grundschule (1–4), Klasse 5–8, Klasse 11–13, Universität (aktuell, noch nicht abgeschlossen); höchster Abschluss: Ausbildung	Ohne festes Beschäftigungsverhältnis
IP_3	Männlich	Syrien	33	9	Mit seiner Frau in einer Wohnung	Abitur	In einem Beschäftigungsverhältnis, ohne nähere Angabe
IP_4	Männlich	Syrien	28	8	Haushalt mit Frau	Abitur	In einem Beschäftigungsverhältnis, ohne nähere Angabe
IP_7	Weiblich	Nordirak, kurdisch	57	25	Zweipersonenhaushalt mit Ehefrau; 2 Kinder (ausgezogen)	Bachelor Architektur in Irak (beendet 1986); nicht anerkannt in D.; Umschulung in D	In einem Beschäftigungsverhältnis – Bedienen von computergesteuerten Maschinen im Produktionsprozess
IP_8	Weiblich	Nordirak, kurdisch	30	25	Lebt mit Mutter und Bruder zusammen	Abitur; derzeit Studentin im letzten Semester (Staatsexamen)	Studentin & Teilzeitarbeit auf kommunaler Ebene
IP_9	Männlich	Syrien, kurdisch	36	9	Wohnt alleine	Klasse 11–13; Abschluss der Sekundärstufe	Besitzer von einem Imbiss; Arbeitet mit seinem Bruder dort
IP_11	Weiblich	Syrien, kurdisch	37	2	Ja, mit Ehepartner & 1 Kind	Hochschulabschluss, Bachelor in Englisch	Ohne festes Beschäftigungsverhältnis
IP_12	Männlich	Syrien, kurdisch	28	2	Ja, mit Ehepartner & 1 Kind	Bachelorabschluss in Elektro- und Computertechnik	In einem Beschäftigungsverhältnis, ohne nähere Angabe
IP_13	Weiblich	Libanon	19	7	Keine Angabe	Realschulabschluss (derzeit Weiterqualifikation Hochschulreife)	Keine Angabe
IP_14	Weiblich	Nordirak, kurdisch	54	24	Keine Angabe	Lehrerin	In einem Beschäftigungsverhältnis – Angestellte im Büro
IP_15	Männlich	Syrien, arabisch	20	5	Wohnt mit Eltern zusammen	Realschulabschluss (derzeit Weiterqualifikation Abitur)	In einem Beschäftigungsverhältnis – geringfügige Beschäftigung Einzelhandel
IP_16	Weiblich	Somalia	22	In Deutschland geboren	Mit Eltern und kleiner Schwester	Fachabitur; derzeit Ausbildung zur Erzieherin/Sozialpädagogin	Ohne festes Beschäftigungsverhältnis (coronabedingt) ansonsten Nebenjob in der Gastronomie
IP_17	Weiblich	Syrien, kurdisch	41	7	Mit beiden Elternteilen	Realschule	Ohne festes Beschäftigungsverhältnis Sie betreut 24 h ihre kranken und alten Eltern
IP_18	Weiblich	Syrien, kurdisch	48	7	Ehepartner & 2 Kinder (Sohn, Tochter)	Abitur	In einem Beschäftigungsverhältnis – Gastgewerbe (seit Corona nur 2‑ bis 3‑mal statt Vollzeit)
IP_19	Weiblich	Irak, arabisch	26	7	Eltern und 3 Brüder (wohnt teilweise bei einer Freundin)	Abitur (derzeit in Ausbildung)	In einem Beschäftigungsverhältnis, ohne nähere Angabe
IP_20	Weiblich	Irak, kurdisch	25	23	Eltern, 2 Geschwister (Bruder, Schwester)	Bachelor in Informatik	In einem Beschäftigungsverhältnis, ohne nähere Angabe
IP_23	Weiblich	Syrien, kurdisch	30	7	Mit Ehepartner und einem Kind	Bachelor-Abschluss in Heimatsland, aktuell: Masterstudentin	Ohne festes Beschäftigungsverhältnis
IP_24	Männlich	Syrien, kurdisch	52	25	Keine Angabe	Abitur	In einem Beschäftigungsverhältnis, ohne nähere Angabe
IP_28	Weiblich	Kasachstan	67	29	Alleine, verwitwet	Keine Angabe	Keine Angabe
IP_33	Weiblich	Irak	31	15	Mit Ehepartner und 2 Kindern	11.–13. Klasse, höchster Abschluss: Fachabitur	In einem Beschäftigungsverhältnis, ohne nähere Angabe
IP_34	Weiblich	Irak	28	20	Mit beiden Elternteilen und 3 Kindern/Geschwistern	Universität	Ohne festes Beschäftigungsverhältnis
IP_35	Weiblich	Syrien, kurdisch	25	8	Mit beiden Elternteilen	Universität; Bachelor an der Universität Bochum	In einem Beschäftigungsverhältnis, ohne nähere Angabe
IP_36	Weiblich	Syrien, kurdisch	58	7	Mit Ehepartner	War Lehrerin von Beruf und hat „Institut“ absolviert	Ohne festes Beschäftigungsverhältnis
IP_43	Weiblich	Deutschland	43	In Deutschland geboren; nie umgezogen	Wohnt mit Kindern zusammen	Fachhochschulreife	In einem Beschäftigungsverhältnis, ohne nähere Angabe
IP_44	Weiblich	Deutschland	48	In Deutschland geboren	Wohnt mit 2 Kindern zusammen (12 & 14 Jahre)	Fachoberschulreife	In einem Beschäftigungsverhältnis – Betreuung der Nachbarin im Pflegedienst, (Kleiderkammer, Hilfestellung bei Dokumenten/Beratung für Flüchtlinge)
IP_45	Männlich	Afghanistan	50	30	Wohnt mit seiner Frau zu zweit zusammen	Abitur	In einem Beschäftigungsverhältnis – geringfügige Beschäftigung Gastronomie

^a^Proband:innen machten Angaben zum Bildungsweg und Abschlüssen in einem Freitextfeld. In der Regel wurde der höchste Bildungsabschluss im ausländischen Schulsystem von den Proband:innen mit dem Abitur gleichgesetzt

## Footnotes

1 Ein Projekt, welches im Rahmen der Innovationsförderung des ehemaligen Bundesministeriums der Justiz und Verbraucherschutz (BMJV, ab Legislatur 2021 BMUV) gefördert und am CERES (Cologne Center for Ethics, Rights, Economics, and Social Sciences of Health) unter dem Namen „Orientierungshilfen im Umgang mit Gesundheitsinformationen im Internet – Konzeptentwicklung und digitale Umsetzung (OriGes I)“ zwischen 2017–2019 entwickelt wurde. Im Folgeprojekt OriGes II „Orientierungshilfen im Umgang mit Gesundheitsinformationen im Internet – Anwendung und Erweiterung in der Praxis“ wird von 2020–2022 die Anwendung und diversitätssensible Weiterentwicklung in der Praxis erforscht und umgesetzt. https://ceres.uni-koeln.de/forschung/projekte/origes (Zugriff: 05.10.2022) und https://ceres.uni-koeln.de/forschung/projekte/origes-ii (Zugriff am: 05.10.2022).

2 Kultursensibilität wird hier verstanden als (An)erkennung dessen, wie stark Online-Gesundheitsinformationen kulturell (von der Mehrheitsgesellschaftskultur in Deutschland) geprägt sind. Erst wenn dies im Sinne eines postmigrantischen Verständnisses von Kultursensibilität (an)erkannt wird, können Gesundheitsinformationen kultur-/diversitätssensibel gestaltet werden.

3 Der hier verwendende Begriff *Community* ist stets heterogen und vielschichtig gedacht. Es gibt Aspekte, welche sich auf die gesamte Community einer sozialräumlichen Nachbarschaft beziehen, während andere Aspekte sich eher auf gewissen Gruppen oder Communities innerhalb einer sozialräumlichen Nachbarschaft beziehen oder gar nur Individuen, welche in der Hustadt leben betreffen.

4 https://www.hs-gesundheit.de/stadtteillabor-hustadt.

5 Der Begriff für diese Dimension des Zugangs wurde in Anlehnung an die Resonanztheorie nach Hartmut Rosa [36] gewählt. In diesem Kontext soll der Begriff auf die Bedeutung einer stimmigen Beziehung zwischen Information und Rezipient:in für die erfolgreiche Auseinandersetzung mit den Inhalten abstellen. Das der Physik entlehnte Konzept der *Resonanz* geht davon aus, dass sich Systeme in wechselseitig Schwingungen versetzen können. Übertragen auf die hiesige Fragestellung, untersuchen die Autor:innen wie und auf welche Weise angebotene Informationen in Rezipient:innen etwas zum Schwingen bringen. Dies geschieht zumeist vorbewusst, so dass für die Entwicklung von diversitätssensiblen Gesundheitsinformationen zunächst einmal von Bedeutung ist, zu erkennen, welche *Schwingungen* durch die angebotenen Informationen erzeugt oder erzeugt werden können.

6 https://www.youtube.com/channel/UC3MqzCB248OyXUDoFzHqI6A, Zugriff: 05.10.2022.

8 Dieses Projekt wird derzeit noch evaluiert. Die Videos können hier eingesehen werden: https://www.gesund-im-netz.net/.
